# A Case of COVID-19-Associated Autoimmune Hemolytic Anemia With Hyperferritinemia in an Immunocompetent Host

**DOI:** 10.7759/cureus.16078

**Published:** 2021-06-30

**Authors:** Zoha Huda, Abdullah Jahangir, Syeda Sahra, Muhammad Rafay Khan Niazi, Shamsuddin Anwar, Allison Glaser, Ahmad Jahangir

**Affiliations:** 1 Medicine, The City University of New York (CUNY) School of Medicine, New York, USA; 2 Internal Medicine, Staten Island University Hospital, Northwell Health, New York, USA; 3 Internal Medicine, Northwell Health, New York, USA; 4 Infectious Disease, Northwell Health, New York, USA; 5 Internal Medicine, Mayo Hospital, Lahore, PAK

**Keywords:** high ferritin, covid-19, autoimmune hemolytic anemia (aiha), hyperferritinemia, hemolytic anemia

## Abstract

We report an interesting case of a middle-aged gentleman who presented with diabetic ketoacidosis (DKA) and tested polymerase chain reaction (PCR) positive for COVID-19 infection. His hospital stay was complicated by acute kidney injury, hematuria, and normocytic anemia. Initial chest x-ray demonstrated bibasilar opacities. D-dimer and C-reactive protein were elevated. During his hospital stay, his hemoglobin decreased from 13.4 g/dL to 9 g/dL, and further workup demonstrated ferritin of 49,081 ng/mL with lactate dehydrogenase of 1665 U/L. He was treated with prednisone and folic acid for autoimmune hemolytic anemia (AIHA). Ferritin was downtrended, and hemoglobin stabilized. As demonstrated by this case report and prior literature review, COVID-19 infection can be associated with AIHA.

## Introduction

Information regarding COVID-19 and associated coagulopathies is rapidly evolving and expanding. Thus far, many cases of autoimmune hemolytic anemia (AIHA) have been reported in patients with comorbidities such as thrombocytopenia, leukemia, lymphoma, various cancers, and autoimmune conditions [1]. The prevalence and significance of AIHA in COVID-19 patients warrant further exploration to elucidate treatment and clinical outlook. We report a case of AIHA and an extremely elevated ferritin level associated with COVID-19 in a patient with no other major risk factors for hemolysis. To our knowledge, this is one of the rare cases of a patient with no underlying malignancy or hematologic dyscrasia presenting with AIHA and COVID-19, and the highest ferritin was reported in this context. This case further supports AIHA as another complication of COVID-19. Additionally, this highlights the importance of laboratory data such as hemoglobin, D-dimer, lactate dehydrogenase (LDH), and ferritin as markers of prognosis and severity of COVID-19 infection [2].

## Case presentation

**Figure 1:**
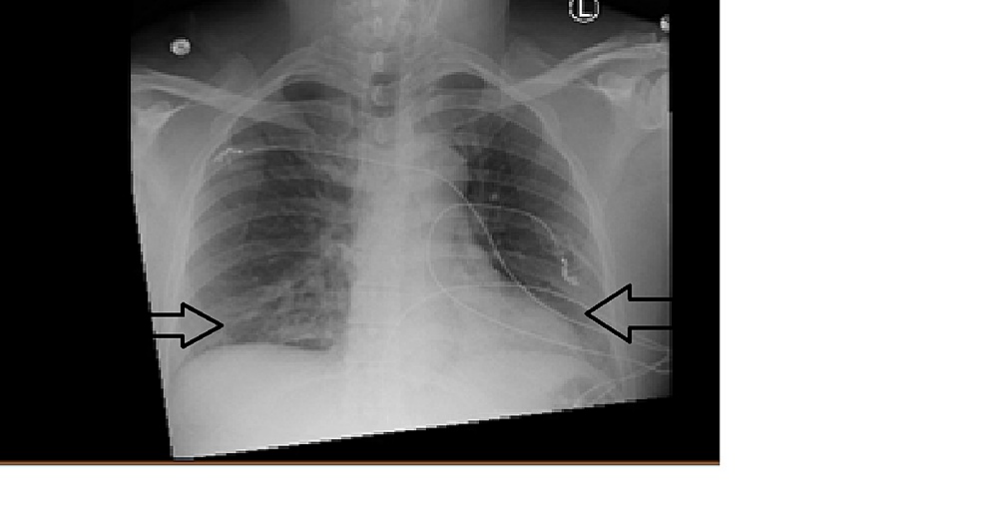
Chest x-ray showing bilateral opacities

A 54-year-old male with a history of uncontrolled diabetes mellitus presented to the emergency room complaining of dyspnea with nausea, vomiting, and polyuria in the preceding days. His COVID-19 rapid testing was positive. The patient was vitally stable and not hypoxemic, thus did not meet the criteria for COVID-19 therapeutics. On admission, glucose was 373 mg/dL, anion gap was 28, carbon dioxide was 7 mEq/L, and b-hydroxybutyrate was > 9 mmol/L. VBG showed partial pressure of carbon dioxide (PCO2) of 22 mmHg and bicarbonate of 8 mEq/L. Chest x-ray showed diffuse bilateral opacities (Figure 1).

He was subsequently admitted for diabetic ketoacidosis, and his hospital stay was complicated by acute kidney injury, hematuria, and AIHA.

On day 8 of hospital admission, microhematuria was noted with urinalysis demonstrating 28 red blood cells per high power field (RBC/HPF). Hemoglobin levels declined from 13.4 g/dL on presentation to 9.0 g/dL while C-reactive protein (CRP) increased to 3.7 mg/L from 0.44 mg/L. D-dimer trended from 226 to 373 ng/mL. The renal sonogram was negative for stones and hydronephrosis. Initial ferritin increased from 3,343 to 49,081 ng/mL on day 6, and further workup demonstrated a positive direct Coombs for IgG on day 9, reticulocyte count of 1.9% with an absolute reticulocyte count of 53.6 K/uL, total bilirubin of 1.4 mg/dL, creatine kinase of 248 U/L, ESR of 90 mm/hr, haptoglobin of 30 mg/dL, and LDH of 1665 U/L (Table 1).

**Table 1:** Hematology panel and inflammatory markers throughout hospitalization (reference ranges added for the average adult male) WBC, White blood cells; CRP, C-reactive protein; AST, aspartate aminotransferase; ALT, alanine transaminase; LDH, lactate dehydrogenase.

Hematology	Day 1	Day 2	Day 3	Day 4	Day 5	Day 6	Day 7	Day 8	Day 9	Day 10	Day 11	Day 12
Hemoglobin (13.5-17.5 g/dL)	13.4	12.3	11.4	11.6	12	11.9	11.2	9.9	9.4	9.0	9.1	8.1
WBC (4-11 K/uL)	9.95	7.83	5.27	3.77	4.85	6.26	7.91	9.92	10.45	9.77	10.33	16.1
Platelets (150-450 K/uL)	206	165	188	168	148	177	210	274	291	365	457	541
CRP (<10 mg/L)	0.44		0.71			3.06		3.84	3.70			
Haptoglobin (41-165 mg/dL)								41	30			
Procalcitonin (<0.15 ng/mL)	0.11		0.09		0.13	0.15		0.15				
Ferritin (24-336 ng/mL)		3343				49,081 (repeated)			27,109 (repeated)			
LDH (140-280 u/L)	172					1780			1665			
D-dimer (<0.5 ng/mL)		226	293		3661				396	343		
Bilirubin (total) (1-1.2 mg/dL)	0.7	1	1.2						1.6	1.4	1.3	1
AST (5-40 u/L)	28	24	25							254	143	98
ALT (7-56 u/L)	28	25	22							79	57	54
Reticulocyte count (0.5%-1.5%)								1.5		1.9		

Vitamin B12, folate, and ultrasound of the spleen and liver were normal. The patient was negative for hepatitis A/B/C, HIV, and cytomegalovirus antibodies. Immune-mediated hemolysis was suspected due to anemia, direct Coombs positivity, low haptoglobin, and elevated LDH. Prednisone 80 mg and 1 mg of folic acid were started on day 7 of hospitalization (weight-based). Ferritin trended down to 27,109 and hemoglobin stabilized. Hyperferritinemia was attributed to diabetic ketoacidosis, COVID-19, and acute kidney injury. There was no suspicion of hemophagocytic lymphohistiocytosis. The patient was discharged home with no complications.

## Discussion

AIHA can be idiopathic or secondary to other autoimmune disorders, malignancies, and infections [3]. Some studies have demonstrated AIHA in COVID-19-positive patients with underlying malignancies or autoimmune disorders [4-12]. However, autoimmune complications because of COVID-19 in patients without associated comorbidities remain to be elucidated. Few studies have noted that AIHA develops in COVID-19-positive patients with no autoimmune disorder or malignancy history. One case of COVID-19 and unprovoked methemoglobinemia has been reported recently [13].

AIHA is an acquired rare condition that develops when autoantibodies develop against self-antigens on the RBCs, which leads to their destruction by the reticuloendothelial system or complement-mediated cell destruction. Cytomegalovirus, Epstein-Barr virus, Coxsackie virus, Parvovirus B19, Hepatitis C virus, mycoplasma, and several concurrent lymphoproliferative disorders can provoke AIHA. The exact mechanism behind this viral-induced cell destruction is unknown, but in most of the literature, it is attributed to molecular mimicry, which leads to self-reactive lymphocytes and unresponsiveness to self-antigens [14,15]. Therefore, after excluding lymphoproliferative disorders, we can proclaim virus-induced AIHA as a cause behind anemia in our case.

Some common laboratory findings in AIHA include anemia, which can be subtle in mild hemolysis. Platelet count and white blood cell count are usually within normal limits; counts can be depressed in some cases of bone marrow suppression from viral infection. Peripheral smear is remarkable for red cell agglutination and spherocytosis. Reticulocyte count and index are increased as a normal bone marrow response to hemolysis. Sometimes reticulocytosis is not evident due to bone marrow disorder or suppression from sepsis. Haptoglobin is decreased, and LDH is elevated; however, LDH may also be elevated as an inflammatory marker from sepsis in COVID-19 infection with lung involvement. This overlap can make it difficult to discern the cause. Urinalysis is positive for blood but negative for erythrocytes. The positive direct antiglobulin test (DAT) represents attachment of complement C3D and immunoglobulins IgG, IgM, IgA to erythrocyte membrane [16]. Ferritin itself is an inflammatory marker, and a previous study shows that in patients with AIHA, a highly significant difference is present in the ferritin levels among patients with hemolysis compared to those in remission [17].

Further investigation regarding the possibility that COVID-19 may cause AIHAs is needed. Of documented cases, AIHA manifested around a median of eight to nine days [18]. Labs may show increased reticulocyte count, LDH, decreased haptoglobin, and indirect bilirubinemia. Complete blood count, D-dimer, coagulation studies, CRP, and ferritin are also important to monitor in patients with COVID-19 [10]. This case demonstrates the highest recorded ferritin level in the context of a COVID-19-positive patient with anemia thus far. In particular, elevated D-dimer, LDH, ferritin, and decreased hemoglobin have correlated to a poorer prognosis of COVID-19 [2]. Anemia was initially presumed to be due to acute illness, specifically dilutional anemia secondary to diabetic ketoacidosis. Therefore, it is important to recognize AIHA as a potential sequela of COVID-19 and to monitor positive patients in the future to hasten treatment and predict the clinical course potentially. A systemic review by Taherifard et al. (2021) highlighted the association of COVID-19 with autoimmune anemias with stress on testing for COVID-19 in sudden anemias in appropriate clinical settings [19].

In terms of treatment, glucocorticoids remain the first-line treatment for AIHA in these patients. Treatment should be initiated with the dose of 1 mg/kg/day prednisone orally or methylprednisolone intravenously [20]. This initial dose is continued until hemoglobin is increased to 10 mg/dl or by 30% of the initial value. If this goal is not achieved after the treatment with steroids within three weeks, other treatment options are considered because there are higher chances of treatment failure. Rituximab and splenectomy are the only second-line treatments with proven efficacy [21]. There are case reports of the use of rituximab in COVID-19, but the decision is complex since rituximab suppresses humoral immunity, which leads to an inability to develop antibodies against the COVID-19 virus [22].

## Conclusions

The association between AIHA and COVID-19 remains to be elucidated. In many cases, AIHA occurred in COVID-19-positive patients with a history of malignancies or autoimmune comorbidities. However, more research is required to examine the link between COVID-19 and AIHA in patients with minimal or no comorbidities.
